# Comfortable Meals in an Institution—Breakfast

**Published:** 1912-06-29

**Authors:** 


### INSTITUTIONAL HOUSEKEEPING.

### Comfortable Meals in an Institution—Breakfast.

There is one inevitable condition attached to all institutional life—the sacrifice of individual tastes. When fifty or a hundred people are fed as one, and have no choice but to eat what is set before them, and when monotonous feeding continues without variety for a whole yearly round, save for the short annual break of a holiday, the body certainly suffers, however healthily adaptable it may be. As no two leaves on a tree are exactly counterparts, so is it true that no two human bodies are alike in needs and requirements. Every character differs, every mind, and also every stomach. And this is much more strongly evident in persons of riper age, who come together from varying environments, with diverse inculcated habits, and special peculiarities; they are very apt to find it irksome to keep a hard-and-fast rule as to diet, however amiable and well disposed they may be. Many, it is true, who come to reside in an institution find themselves better provided for than ever in their lives before. We know well how some homes are managed—the irregular meals, the ignorance of "food value," the bad cooking, the mistaken ideas concerning cheapness, the self-indulgence, late hours, and other defects, which tend to impair health and digestion. After living under such conditions numbers find an astonishing benefit from the wholesome and regular institutional dietary. But there are many others who have lived in well-ordered homes and are accustomed to an appetising variety in their food and a healthful choice of what suits them best. To these it is exceedingly galling to find a daily *menu* restricted within the narrowest limits, and to be obliged to accept without alternative that which they have found unsuitable and have steadily refused for years.

### BREAKFAST IDIOSYNCRASIES.

At breakfast, a number of women assemble at the sound of the bell, and to each one is dealt out a cup of tea or coffee. It may (or may not) have been carefully prepared, hot, and not too strong, and there may be sufficient milk and sugar, in many small jugs and basins, ready to hand, without delay. But to some people tea or coffee are quite injurious unless much diluted with hot milk or water, and on the ordinary breakfast table this is omitted. It would be very easy to supply hot milk and water, it could be passed down the table in covered jugs, and most people would gladly accept it, but the matron probably considers this small accessory in the light of "a modern fad." Next comes generally a plateful of bacon or ham, or meat in some other form, a delicious luxury to some, but to others an indigestible impossibility so early in the day, and they pass it by. Why may not a watchful eye note those who refuse the substantial dish, and ask them in private what they prefer in its place? It is not right they should undertake hours of laborious toil without good nourishment. An egg or a plate of porridge would not be costly, and might make all the difference to a delicate appetite. In every case the staple food at breakfast consists of bread, and to that article far too little attention is paid. Bread should not be exposed to the air by being placed on the table long before the meal is ready. There should be white bread and brown, and, if possible, bread from various bakers occasionally, making a pleasing variation in the monotony of the first meal. Butter should be placed on the table in plain slabs on many little platters, and should never be so cheap as to be nasty.

Nor is it an utter impossibility to include toast as an item on the institutional breakfast table. In any well-appointed kitchen toast can be made quickly in large quantities, and what is left is always useful to be cut up into dice for soup, or to be put round dishes of hash or under vegetables. Syrup, jam, or marmalade is indispensable, yet often omitted. The craving for sweet things is a healthy one, and far too little considered in institutional life.

It should be borne in mind continually that variety in food conduces to good temper and loyalty of spirit, and eases many of the burdens of a hard-working life. If we awoke every morning to the same kind of weather we should feel disgusted and weary, and a fresh sensation to the palate is as agreeable as a sunny day after a week of London fog.

---

### Housekeeping Queries.

"Nemo" *writes*: "*I shall be much obliged if you can give the usual allowance of rations per head in a small hospital.*"

It is very unusual at the present day to have any settled allowance. It has long been found more economical to provision the household as a whole, instead of setting aside so much per head. But it is very important to divide up the amount spent each week by the number of inmates boarded, and thus ascertain any fluctuations in cost. Moreover, in hospitals, however small, the patients must not be reckoned in with the staff, or all control over the cost of the rations is lost. The cost can be calculated roughly as follows: Suppose you have an average of twenty-five patients in the hospital; you may reckon 3s. 6d. a week for each, those on milk diet costing rather less, those on convalescent diet costing more. (But this does not allow for "extras.") The twenty-five patients then will cost about £4 7s. 6d. For this number of patients you will have eight or ten nurses, including yourself, and three or four servants to board, say, twelve altogether. It is hardly possible in a small household to make any difference in the fare provided for the separate tables, and it may be reckoned that a skilful manager could provide an ample and varied diet in any locality where prices are reasonable for 6s. 6d. a head. You must then add on £3 18s. to your estimate for the patients' board, and you will have a weekly total of £8 5s. 6d. for the food for the whole establishment. It is quite easy for the matron to work out the figures for an institution of any size on these lines. If the amount does not exceed 3s. 6d. per head for each patient and 6s. 6d. per head for each member of the nursing and domestic staff, you may be satisfied that you are not spending too much. But it is far better to keep the bills for the patients entirely distinct, and reckon out each week the exact cost of each section of the household, instead of working them in one with another.

---

#### To Our Readers.

These columns are open to queries and discussion touching all domestic matters. Matrons are invited to send us the result of any experiments they are making with a view to promoting the comfort of the staff. Estimates given of the cost of boarding patients, nurses, and staff.

